# The Burden of the “False‐Negatives” in Clinical Development: Analyses of Current and Alternative Scenarios and Corrective Measures

**DOI:** 10.1111/cts.12478

**Published:** 2017-07-04

**Authors:** T Burt, KS Button, HHZ Thom, RJ Noveck, MR Munafò

**Affiliations:** ^1^ Burt Consultancy LLC. Durham North Carolina USA; ^2^ Department of Psychology University of Bath UK; ^3^ School of Social and Community Medicine University of Bristol Bristol UK; ^4^ Department of Medicine, Division of Clinical Pharmacology Duke Clinical Research Unit Durham North Carolina USA; ^5^ MRC Integrative Epidemiology Unit, UK Centre for Tobacco and Alcohol Studies, School of Experimental Psychology University of Bristol UK

## Abstract

The “false‐negatives” of clinical development are the effective treatments wrongly determined ineffective. Statistical errors leading to “false‐negatives” are larger than those leading to “false‐positives,” especially in typically underpowered early‐phase trials. In addition, “false‐negatives” are usually eliminated from further testing, thereby limiting the information available on them. We simulated the impact of early‐phase power on economic productivity in three developmental scenarios. Scenario 1, representing the current *status quo*, assumed 50% statistical power at phase II and 90% at phase III. Scenario 2 assumed increased power (80%), and Scenario 3, increased stringency of alpha (1%) at phase II. Scenario 2 led, on average, to a 60.4% increase in productivity and 52.4% increase in profit. Scenario 3 had no meaningful advantages. Our results suggest that additional costs incurred by increasing the power of phase II studies are offset by the increase in productivity. We discuss the implications of our results and propose corrective measures.

**Study Highlights**

**WHAT IS THE CURRENT KNOWLEDGE ON THE TOPIC?**

✓ Early‐phase clinical development studies are usually underpowered, with little knowledge about the extent, magnitude, and economic impact of the consequent “false‐negatives.” Only one brief previous report, using different methodology, has studied the topic.^48^

**WHAT QUESTION DID THIS STUDY ADDRESS?**

✓ Our simulations aimed to study the impact of statistical error thresholds on clinical development productivity.

**WHAT THIS STUDY ADDS TO OUR KNOWLEDGE**

✓ Underpowered phase II studies result in unacceptably high rates of “false‐negatives.” The burden of “false‐negatives” on clinical development productivity is potentially enormous, leading to loss of effective treatments and associated commercial profits. Increasing the power of early‐phase trials is worth the investment in larger sample sizes.

**HOW THIS MIGHT CHANGE CLINICAL PHARMACOLOGY OR TRANSLATIONAL SCIENCE**

✓ Increasing power of early‐phase clinical trials could improve productivity of drug development with increased profits due to reduction in frequency of “false‐negatives” compensating the costs of larger sample‐sized studies.

Clinical development is increasingly a complex, risky, lengthy, failure‐prone, and costly process with considerable healthcare benefits and commercial profits at stake.^1^, ^2^, ^3^, ^4^ Contributing to the costs and delays are statistical errors that lead to “false‐positive” and “false‐negative” results. The “false‐positives” are the treatments that appear promising but in fact are not. These errors can lead to expensive follow‐up testing, exposure to unnecessary risks and ineffective treatments, and potentially costly delays in development of promising back‐up treatments. The “false‐negatives” are the effective treatments wrongly eliminated, leading to missed healthcare and economic opportunities and are the subject of our investigation.

While the “falseness” of the “false‐positives” may be exposed in adequately powered, larger confirmatory trials, the burden of the “false‐negatives” is mostly hypothetical, with little empirical evidence to characterize it and guide corrective measures.^5^ This is because the “negatives” usually exit the developmental process and are not exposed to future adequately powered confirmatory trials. To establish a better understanding of the “false‐negatives” in clinical development, we studied them in several traditional and alternative simulated scenarios. We were interested in two questions. First, how does the proportion of “effective” treatments that ultimately succeed (i.e., pass at phase II and phase III) and those that ultimately fail (at either phase II or phase III) change in different scenarios? Second, what are the costs and potential profits across the different scenarios? We present the results for three predefined developmental scenarios (Scenarios 1–3) and a fourth scenario that emerged as optimal from follow‐up analyses (Scenario 4).

## MATERIALS AND METHODS

To answer the first question, we created a hypothetical general scenario whereby 100 potential treatments enter at phase II, with those determined successful (i.e., the “positives”) proceeding through to phase III. We assumed that 25% of these are “effective” treatments and 75% are “ineffective” treatments.^2^, ^6^, ^7^, ^8^, ^9^ Scenario 1 (“*Status quo*”) uses the typical values for Type‐I and Type‐II error rates currently in use in treatment development. The Type‐I error rate (α) is set at 5% for phase II and at 0.25% for phase III, given the regulatory requirement that treatments show efficacy in two independent trials at phase III. The Type‐II error rate (β) is set at 10% for phase III trials, representing 90% statistical power on average, and at 50% for phase II, representing 50% statistical power (1‐β) on average.^10^ (see detailed discussion of the assumptions in the **Supplemental Information**). In Scenario 2, phase II has 80% power. In Scenario 3, the significance threshold is more stringent (1%) in phase II. Separately, we searched the space of alpha and beta thresholds to identify the optimal combination of alpha and beta in terms of developmental productivity (Scenario 4).

To answer the second question regarding costs and potential profits in each of the scenarios, we assumed cost per study in Scenario 1 of $40M for phase II studies and $163M for phase III studies.^2^ Costs of phase II in Scenarios 2–4 increased proportionally to sample size but with a conservative 80% correction due to an economies of scale reduction in cost‐per‐participant at higher sample sizes. We also assumed a return on a single successful treatment of $2,500M, based on estimates of the costs of taking a treatment through the development process^2^, ^7^, ^11^ and the need for developers to have a return on their investments.

In addition, we conducted sensitivity analyses to explore greater effect sizes at phase II. These “adjusted” analyses (**Supplemental Information Additional Analyses, Table C.2**) were designed to account for the potential use of surrogate end points and/or enriched samples at phase II trials that may result in greater effect size when compared with the clinically relevant end points and/or nonenriched patient populations usually used at phase III. We also explored the impact of a different percentage of “effective” treatments entering phase II (10% instead of 25%). Finally, probabilistic sensitivity (Monte Carlo) analyses were conducted to test the robustness of our conclusions to variations in input parameters. These analyses varied the effect size, the proportion of effective treatments, the costs per patient, and the return on success. The alpha and beta levels of the scenarios were held fixed (**Supplemental Information Additional Analyses**).

## RESULTS

The number of “effective” and “ineffective” treatments that pass and fail testing at phase II and phase III in each of the four scenarios is shown in **Table** 1. Those that pass at phase II carry on to phase III, whereas those failing at phase II are removed from the pipeline. Those “effective” treatments which pass at phase II and phase III are described as *true‐positives* (i.e., successful treatments), while those “effective” treatments that fail (at either phase II or phase III) are described as *false‐negatives* (i.e., missed opportunities). The “ineffective” treatments which pass at phase II and phase III are described as *false‐positives* (i.e., incorrectly identified as effective), while those that fail at either phase II or phase III are described as *true‐negatives* (i.e., correctly identified as ineffective). These are shown in **Table** 2, together with the cost estimates for phase II and phase III, and the likely profit under each scenario.

**Table 1:** Passage of “good” and “bad” treatments through the development pipeline

**Scenario 1: *Status Quo***							
	**Phase II (α = 5%; 1‐β = 50%)**			**Phase III (α = 0.25%; 1‐β = 90%)**			
**Total treatments**	***N =* 100 (100%)**			***N =* 16.3 (100%)**			
Good treatments	*N =* 25 (25%)	Pass	12.5	*N =* 12.5 (77%)	Pass		10.1
		Fail	12.5		Fail		2.4
Bad treatments	*N =* 75 (75%)	Pass	3.8	*N =* 3.8 (23%)	Pass		0.0
		Fail	71.3		Fail		3.7

**Scenario 2: High power at phase II**							
	**Phase II (α = 5%; 1‐β = 80%)**			**Phase III (α = 0.25%; 1‐β = 90%)**			
**Total treatments**	***N =* 100 (100%)**			***N =* 23.8 (100%)**			
Good treatments	*N =* 25 (25%)	Pass	20.0	*N =* 20 (84%)	Pass		16.2
		Fail	5.0		Fail		3.8
Bad treatments	*N =* 75 (75%)	Pass	3.8	*N =* 3.8 (16%)	Pass		0.0
		Fail	71.3		Fail		3.7

**Scenario 3: Stringent Alpha**							
	**Phase II (α = 1%; 1‐β = 50%)**			**Phase III (α = 0.25%; 1‐β = 90%)**			
**Total Treatments**	***N =* 100 (100%)**			***N =* 13.3 (100%)**			
Good Treatments	*N =* 25 (25%)	Pass	12.5	*N =* 12.5 (94%)	Pass		10.1
		Fail	12.5		Fail		2.4
Bad Treatments	*N =* 75 (75%)	Pass	0.8	*N =* 0.8 (6%)	Pass		0.0
		Fail	74.3		Fail		0.7

**Scenario 4: Lenient alpha and higher power at phase II**							
	**Phase II (α = 20%; 1‐β = 95%)**			**Phase III (α = 0.25%; 1‐β = 90%)**			
**Total treatments**	***N =* 100 (100%)**			***N =* 38.8 (100%)**			
Good treatments	*N =* 25 (25%)	Pass	23.8	*N =* 23.8 (61%)	Pass		19.2
		Fail	1.3		Fail		4.5
Bad treatments	*N =* 75 (75%)	Pass	15.0	*N =* 15 (39%)	Pass		0.0
		Fail	60.0		Fail		15.0

**Figure 1:**
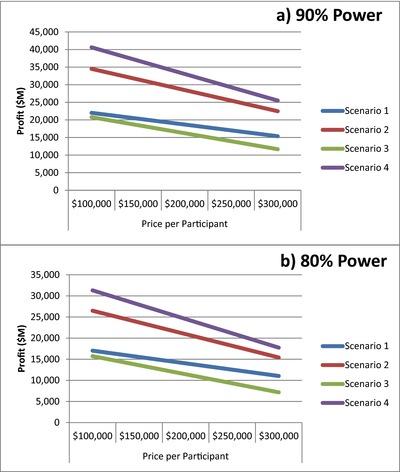
Impact of cost per participant on net profit. A range of costs are explored in terms of their impact on net profit in each of the four scenarios. (**a**) and (**b**) indicate 80% and 90% power at phase III, respectively. The current cost per participant ($200,000) is derived from the average cost of phase II program ($40M) 2 divided by the average number of participants in phase II studies (*N =* 200).

Scenario 1, “*Status Quo*” represents the current, reference situation where the power of phase II (50%) is substantially lower than phase III (90%). In Scenario 2, “High Power at phase II” phase II has 80% power. In Scenario 3, “Stringent Alpha,” the significance threshold is more stringent (1%) in phase II. Scenario 4, “Lenient Alpha and Higher Power at phase II” alpha is set at 20% and the power is 95%. All four scenarios assume 25% of treatments that enter phase II are “good” and 75% “bad.” The number of treatments that enter phase III is determined by phase II alpha and beta error thresholds. The percentage of “good” and “bad” treatments entering phase III differs by scenario but since they are calculated against the overall number of treatments that enter phase III they always total 100%. For example, in Scenario 1, the number of “good” treatments, 12.5, is the number that made it through phase II (50% of 25 treatments entering phase II) and constitutes 77% of the total 16.3 treatments that enter phase III in this scenario. The overall number of “true” and “false” treatments passing through both phases is depicted in **Figure 1**.

Scenario 1: Low power (50%) at phase II, high power at phase III (90%); Scenario 2: High power (80%) at phase II (alpha as in Scenario 1); Scenario 3: Stringent alpha (1%) at phase II (power as in Scenario 1); Scenario 4: Lenient alpha (20%) and higher power (95%) at phase II.

**Table 2:** Cost analysis of “effective” and “ineffective” treatments entering the development pipeline

Scenario 1: *Status Quo*								
	**Phase II**	**Cost ($M) unadjusted**	**Cost ($M) adjusted**	**Phase III**	**Cost ($M)**	**Total**	**Profit ($M) unadjusted**	**Profit ($M) adjusted**
True positives	12.5	4,017 (2,009 to 5,958)	2,011 (1,008 to 3,001)	10.1	2,650 (1,197 to 4,477)	10.1	18,608 (4,906 to 37,242)	20,614 (7,143 to 39,020)
False negatives	12.5			2.4		14.9		
False positives	3.8			0.0		0.0		
True negatives	71.3			3.7		75.0		

Scenario 2: High power at phase II								
	**Phase II**	**Cost ($M) unadjusted**	**Cost ($M) adjusted**	**Phase III**	**Cost ($M)**	**Total**	**Profit ($M) unadjusted**	**Profit ($M) adjusted**
True positives	20.0	8,119 (4,050 to 12,046)	4,017 (2,009 to 5,958)	16.2	3,100 (1,359 to 5,376)	16.2	29,208 (7,068 to 59,281)	33,310 (11,619 to 62,920)
False negatives	5.0			3.8		8.8		
False positives	3.8			0.0		0.0		
True negatives	71.3			3.7		75.0		

Scenario 3: Stringent alpha								
	**Phase II**	**Cost ($M) unadjusted**	**Cost ($M) adjusted**	**Phase III**	**Cost ($M)**	**Total**	**Profit ($M) unadjusted**	**Profit ($M) adjusted**
True positives	12.5	6,938 (3,471 to 10,287)	3,470 (1,739 to 5,180)	10.1	1,730 (708 to 3,120)	10.1	16,589 (2,501 to 35,511)	20,056 (6,425 to 38,625)
False negatives	12.5			2.4		14.9		
False positives	0.8			0.0		0.0		
True negatives	74.3			0.7		75.0		

Scenario 4: Lenient alpha and much higher power at phase II								
	**Phase II**	**Cost ($M) unadjusted**	**Cost ($M) adjusted**	**Phase III**	**Cost ($M)**	**Total**	**Profit ($M) unadjusted**	**Profit ($M) adjusted**
True positives	23.8	8,802 (4,390 to 13,029)	4,326 (2,157 to 6,415)	19.2	5,051 (2,428 to 8,088)	19.2	34,219 (7,651 to 70,138)	38,695 (12,670 to 74,080)
False negatives	1.3			4.5		5.8		
False positives	15.0			0.0		0.0		
True negatives	60.0			15.0		75.0		

Scenario 1: Low power (50%) at phase II to high power at phase III (90%); Scenario 2: High power (80%) at phase II (alpha as in Scenario 1); Scenario 3: Stringent alpha (1%) at phase II (power as in Scenario 1); Scenario 4: Lenient alpha (20%) and higher power (95%) at phase II.

Under Scenario 1, 16.3% of treatments are successful at phase II and enter phase III (12.5% “effective,” representing 50% of the original “effective” entering phase II, plus 3.8% “ineffective” treatments). This means that 77% (12.5 of 16.3) of the treatments entering phase III are in fact “effective” treatments and 61.9% (10.1 of 16.3) will pass at phase III, of which the vast majority (99%; 10 of 10.1) will be “effective” treatments. However, in this scenario only 10.1 of the original 25 (i.e., 40.4%) “effective” treatments pass at both phase II and phase III, with 12.5 being lost at phase II and 2.4 being lost at phase III for a total 14.9 “false‐negatives.”

Under Scenario 2, 23.8% of all treatments pass at phase II (20% “effective” treatments plus 3.8% “ineffective” treatments). This means that 84.0% of treatments entering phase III are “effective” treatments. Of these, 68.1% will pass at phase III, with the vast majority of them “effective” treatments. Critically, in this scenario 16.2 of the original 25 “effective” treatments (i.e., 64.8%) pass at both phase II and phase III, with 5.0 lost at phase II and 3.8 lost at phase III for a total 8.8 “false‐negatives.” This represents a 60.4% increase in productivity over Scenario 1 (from 40.4% to 64.8%) and a reduction from 59.6% to 35.2% in the proportion of “false‐negatives” (i.e., the “missed opportunities”). While the cost of Scenario 2 is considerably greater than Scenario 1, being $8,163M at phase II and $3,868M at phase III (104.1% and 46.2% increase vs. Scenario 1, respectively), the number of successful treatments would return $40,523M, representing a profit of $28,492M and an overall 52.4% increase in profit vs. Scenario 1.

Under Scenario 3, 13.3% of all treatments pass at phase II, the vast majority being “effective” treatments because the stringent Type‐I error rate almost completely removes “ineffective” treatments from the discovery pipeline. This means that 94% of the treatments entering phase III are “effective” treatments. Of these, 75.9% will pass at phase III, essentially all being “effective” treatments. However, as in Scenario 1, only 10.1 of the original 25 “effective” treatments (i.e., 40.4%) pass at both phase II and phase III, meaning there was no increase in productivity. Based on our cost estimates, the cost of Scenario 3 would be $6,939M in phase II, but only $2,158M at phase III (73.5% increase and 18.4% reduction vs. Scenario 1, respectively). While the successful treatments would return $25,317M, almost exactly as in Scenario 1, this would represent a profit of only $16,221M (reduction of 13.2% vs. Scenario 1) given the higher study costs overall and no improvement in the proportion of “missed opportunities” (the *false‐negatives*).

Exploring the space of alpha and beta allowed the identification of Scenario 4 with optimal combination of both (i.e., even lower (5%) beta but more lenient (20%) alpha at phase II than the other scenarios), 38.8% of all treatments pass at phase II. Of these, 19.2 (49.5%) will pass at phase III, essentially all being “effective” treatments, representing 76.8% of the original 25 “effective” treatments, and constituting the highest productivity of the four scenarios. When compared with Scenario 1, the *status quo*, the increase in productivity is 90.1% (from 40.4% to 76.8%). While the cost of Scenario 4 would be considerably greater than Scenario 1, being $8,816M at phase II, and $6,311M at phase III (120.4% and 138.5% increase vs. Scenario 1, respectively) the number of successful treatments would return $48,188M, representing a profit of $33,060M and an overall 76.9% increase in profit vs. Scenario 1.

In **Figure** **1** we show the net profit under the three scenarios we describe, and the impact of a range of costs per participant. The current cost per participant ($200,000) is derived from the average cost of phase II program ($40M)^2^ divided by the average number of subjects in phase II studies (*N =* 200). In all cases, Scenario 4 (lenient alpha and higher power at phase II) performs the strongest, even when the cost per participant is doubled.

**Figure 2:**
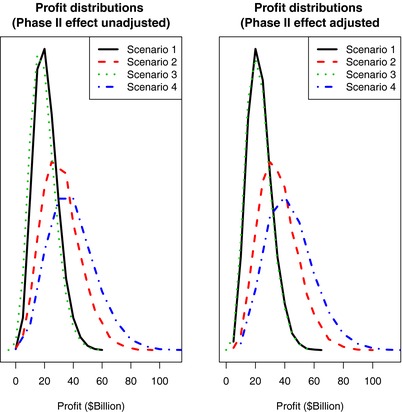
Distribution of simulated profits from probabilistic sensitivity analysis for the four scenarios varying cost‐per‐participant, effect size, proportion of “effective” treatments, and expected returns. The figure demonstrates that while Scenarios 2 and 4 are superior, the overlapping distribution indicates a degree of uncertainty.

**Figure 3:**
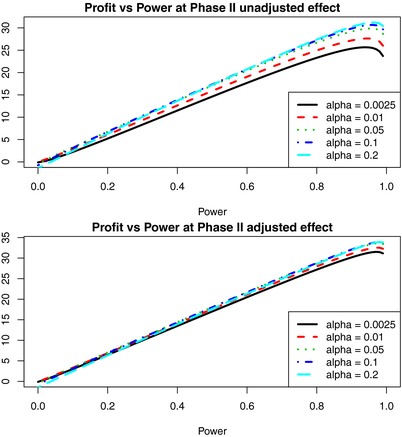
Plot of profit against power for a range of alphas at phase II. All lines assume two phase III trials with alpha of 5% and power of 90% each. Plotting lines with higher phase II alphas show that alpha of 20% (light blue) is a maximum, and optimal power is 95%, which corresponds to Scenario 4. An alpha of 5% (green) corresponds to Scenarios 1 and 2 while an alpha of 1% (red) corresponds to Scenario 3. Power has relatively more influence on profit than alpha over the range of these parameters but it is worth noting the influence of alpha increases as power increases.

Probabilistic sensitivity (Monte Carlo) analysis provided results based on 10,000 samples of 100 candidate drugs. These are presented in **Figures** **2** and **3** (and **Tables B, C in Supplemental Information**). Scenario 4 has the highest successful treatments, fewest missed opportunities, and highest profits. Our conclusions hold whether or not the effect sizes are adjusted up at phase II to account for use of potent surrogate end points and/or “enriched” populations more likely to respond to the therapeutic intervention (**Table** 2 **; Table C.2, Supplemental Information**). In fact, even “unadjusted” alternative Scenarios 2 and 4 are more cost‐efficient than the “adjusted” “*status quo*” Scenario 1. We also explored correlations between simulated differences in profits under competing scenarios and sampled input parameters. This indicated a clear correlation between difference in profits and both return on investment and proportion of “effective” treatments, and these maximized the superiority of Scenarios 2 and 4 over the *status quo* of Scenario 1 (see **Figures A, B, Supplemental Information**).

## DISCUSSION

We simulated three scenarios to study the impact of Type‐I and Type‐II statistical errors on the productivity of staged clinical development. While traditional Scenario 1 appears optimized to remove “ineffective” treatments at phase II, this is done at the expense of also losing half of all “effective” treatments at this stage. A more profitable outcome is realized under Scenario 2 by increasing the power of phase II trials from an average of 50% to 80%. While this entails considerably greater investment at phase II, the far greater number of “effective” treatments subsequently retained at phase II that eventually pass at phase III (from 40.4% in Scenario 1 to 64.8% in Scenario 2, a 60.4% increase in productivity) greatly increases the return on this investment. In addition, the higher proportion of “effective” treatments being tested at phase III (84% instead of the current 77%) means more efficient use of resources at this late and expensive stage of development. In Scenario 3, increasing the stringency of the alpha criterion (from 5% to 1%) for a treatment passing phase II would prohibitively increase sample size and associated study costs at phase II but only marginally reduce the costs at phase III.

An exploratory *post‐hoc* Scenario 4 was identified as the optimal scenario, providing the fewest false‐negatives and greatest return on investment. In this scenario, with even higher (95%) power but a more lenient (20%) alpha at phase II, 76.8% of the original 25 “effective” treatments were identified as true‐positives, a 90.1% increase in productivity and 76.9% increase in profit vs. Scenario 1. Scenario 4 may be consistent with the “intuitive” approach at early‐phase underpowered studies to consider “trend” results (i.e., a more lenient alpha).

Probabilistic sensitivity (Monte Carlo) analyses confirmed that our results are maintained under a range of the basic assumptions (namely, effect size, proportion of “effective” treatments at entry to clinical development, developmental costs, and expected returns) (**Supplemental Information**). Furthermore, we conducted a sensitivity analysis that assumed a larger effect size at phase II than at phase III, with the justification being the potential use of more powerful surrogate end points and/or “enriched” samples that recruit patients more likely to respond. We examined whether the higher (“adjusted”) effect size could counter the smaller sample size at phase II. However, the same development strategies were identified as optimal under this sensitivity analysis, so our conclusions appear robust to assumptions of greater effect size at phase II (**Supplemental Information Table C.2 and Figure A**). The “adjusted” scenarios are more cost‐efficient than the respective “unadjusted” ones but, notably, even “unadjusted” Scenarios 2 and 4 are more cost‐efficient than the “adjusted” “*status quo*” Scenario 1 (**Table** 2). There appears to be little impact of effect size on the differences in simulated profits. Importantly, the potential return on investment will be known when designing phase II studies and our analyses suggest that if the expected return is large a high power at phase II is likely to be justified (as was previously suggested by Cartwright *et al*.^5^)

**Figure 4:**
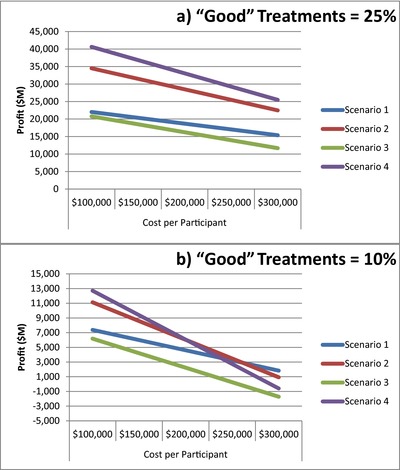
Impact on profitability of percentage of “effective” treatments entering phase II. (**a**) Our main results assume 25% “effective” treatments entering efficacy testing in phase II of clinical development. (**b**) Profits are considerably reduced in all scenarios and the difference between Scenarios 1, 2, and 4 is minimized if the percentage of “effective” treatments entering phase II is 10%.

Finally, we assumed that 25% of treatments entering phase II are “effective” treatments. This proportion is likely to vary considerably across therapeutic areas. Nevertheless, if we instead assume that only 10% of treatments entering phase II are “effective” treatments, then Scenario 2 only outperforms Scenario 1 at relatively low costs per participant, while at higher costs per participant Scenario 1 is optimal (**Figure** **4**). Therefore, in situations where there are very few successful treatments evaluated, the current *status quo* may be the better strategy. Nevertheless, recent literature suggests that only rarely overall success rates are under 10% in clinical development.^2^, ^7^, ^8^

## Power, “false‐negatives,” and implications for clinical development

The proportion of “false‐negatives” is a function of the statistical power of a study; the greater the power the lower the proportion of “false‐negatives.” The power is 1‐beta (the Type‐II error). The Type‐II error is a function of the effect size, sample size, Type‐I error, and expected variability of the sample being tested^12^:

$$Power\;=\;1-\Phi \left[z_{1-\alpha }-\frac{\mu_{1}-\mu_{0}}{\sigma /\sqrt{n}}\right]$$

Where *Φ* is the cumulative standard normal distribution function, *z* is the standardized normal distribution, *α* is the Type‐I error, *n* is the sample size; *σ* is the standard deviation, *μ_1_ − μ_0_* is the difference between the group means, and *μ − μ_0_/σ* is the effect size.^13^

Uncertainty about effect size and variability at early stages of development may give rise to errors in power calculations. In addition, commercial, strategic, and resource considerations may limit sample size. Finally, there are four important asymmetries between the “false‐positives” and the “false‐negatives” that have potential to limit study power and increase the impact of “false‐negatives”:

A) **The Threshold Asymmetry** – In null hypothesis significance testing (NHST), still the cornerstone of most statistical inference, the “false‐negative” (i.e., β or Type‐II error) rate is conventionally set at the β = 10% or 20% level (but is often much higher, especially in underpowered early‐phase drug trials), while the “false‐positive” (i.e., Type‐I error) rate is set at α = 5%.^5^, ^14^ This means that there is an implicit asymmetry in the relative importance ascribed to the two types of error.^15^ With Type‐II error at 20%, this is four times as high as the Type‐I error, but in the case of phase II studies. If, as we assume, power is 50% in the typically underpowered phase II study, then the Type‐II error is 10 times more likely than the Type‐I error. Traditionally, phase II studies are conducted with a smaller sample size, and therefore with even lower power than is implied by the above‐mentioned asymmetry. Typical sample sizes are sometimes an order of magnitude smaller in phase II studies than in phase III (see **Supplemental Information** for more details).^10^, ^12^, ^16^, ^17^
B) **The Developmental Asymmetry** – The clinical development process is one of staged‐development whereby candidates are exposed to successive testing. However, only the “positives” persist in the process of development, and hence are exposed to further testing, while “negatives” are eliminated from further testing. The additional tests (i.e., the larger confirmatory phase III trials) that the “positives” are exposed to sometimes identify them as “false‐positives.”^2^, ^7^, ^8^ The “negatives” of early clinical development, both the “true” and the “false” ones, on the other hand, are eliminated from the developmental process and do not go through the later‐phase “verification” and “validation” process. The result is a skewed body of knowledge: we know more about the “false‐positives” than we know about the “false‐negatives.” Another result is that with successive testing the “false‐negatives” accumulate and increase, while the “false‐positives” are discovered to be false and therefore eliminated and reduced. One reassuring conclusion from our analyses is that the likelihood of “false‐positives” making it through the developmental process is miniscule (about 1 in 1,000 treatments developed) (**Table** 2). However, even with our most productive scenario (Scenario 4), 5.8% of “false‐negative” make it through the clinical development process.
C) **The Economic Asymmetry** – While the cost of a “false‐positive” may be expensive phase III trials at several hundred million dollars at most, the cost of a “false‐negative” may be the loss of a blockbuster worth billions of dollars.
D) **Study Interpretation Asymmetry** – When interpreting study results, attention almost exclusively is directed at the “significance” of the results (for example, for publication purposes) as determined by surpassing the threshold for the Type‐I error, or alpha, usually set at 0.05. However, no less important for assessing the validity of the results is the knowledge of the Type‐II error. A recent review by Pereira *et al*. noted the frequent detection of false large effect in early‐phase studies.^18^ While all were significant at the 0.05 alpha level, their low power exposed them to detection of spurious results.

Is it possible that the traditional thresholds for “alpha” and “beta” of clinical trials are anachronistic, rooted in an era with different healthcare needs, and economic realities? Specifically, does the asymmetry between the traditional alpha and beta thresholds contribute to an inefficient development process by allowing and tolerating greater error in the proportion of “false‐negatives” than that of “false‐positives”? This asymmetry possibly originated in an era when resources were constrained and true hypotheses were easier to confirm (so called “low‐hanging fruit”). Possibly there were enough treatments with large true effects capable of being identified with the smaller sample sizes of phase II studies. Possibly, also, return on investment was not as high, making the large sample sizes required in phase II in our Scenario 2 prohibitive.

However, today we likely have the opposite scenario, where “effective” treatments are more difficult to come by and the stakes and potential rewards are much higher. Regulators, academicians, industry, ethicists, and the public at large have identified the resulting stagnation, inefficiency, and uncertainties of clinical development as a major public health challenge.^19^, ^20^, ^21^, ^22^ In this case, the additional resources required for larger sample sizes may not seem that prohibitive anymore, given that the potential rewards of harvesting as many “true‐positives” as possible are much more attractive. There is also a suggestion that the overall true effect size of new treatments is gradually being reduced in what has been termed “innovation to extinction.”^23^, ^24^ This may mean that new statistical and strategic approaches and tools are required (see under proposed approaches, below).^25^, ^26^, ^27^ It may be telling that our most productive scenario (Scenario 4) reverses the above‐mentioned threshold asymmetry between the Type‐I and Type‐II errors (from α = 5%, β = 50% to α = 20%, β = 5%), suggesting greater value for the missed opportunities of the “false‐negatives” than the excessive testing of the “false‐positives.”

Recent publications attempting to address the underproductivity of clinical development have focused on the high and expensive attrition rates at phase III as drivers of unproductivity.^28^, ^29^ It may therefore seem counterintuitive that we come up with a recommendation that increases the number of compounds reaching phase III. However, under Scenario 2, virtually all this increase (from 12.5 to 20.0 treatments; **Table** 1) is composed of “effective” treatments having been previously eliminated as “false‐negatives” in underpowered phase II studies. In addition, the increase in the costs of phase III studies is more than compensated for by the higher percentage of technical success (60.4% overall increase in productivity). Recommendations to “seek truth, not progression” are to be lauded; however, they appear to be aimed mainly at the “false‐positives” (i.e., when the nontrue progress) 29, 30 Similar efforts should be directed at the “false‐negatives,” as suggested below.

## Preventative, minimizing, and mitigating approaches

We propose the following preventative, minimizing, and mitigating approaches to increase the effective power of early‐phase studies. Although most are currently being used, they are not used universally or in concert:

1. **Increasing sample size**. The most straightforward way to increase study power is to increase its sample size. Our analyses suggest that the increased costs will be rewarded by increased productivity. Increased sample size does not necessarily mean a longer duration of phase of development, as increases in number of sites and speed of recruitment can counter that impact.
2. **Increase effect size**. Effect size is usually thought of as fixed for a given treatment, but in fact it is the average of the effects observed in the test sample. Suboptimal choice of doses and/or target population may “dilute” the maximal effect of the treatment. To address uncertainties regarding the dose–effect relationship in phase II studies and inform optimal dose selection in confirmatory phase III trials, the MCP‐Mod (Multiple Comparison Procedures Modeling) was developed and endorsed by the US Food and Drug Administration (FDA) and European Medicines Agency (EMA).^31^ In addition, identifying and validating biomarkers that narrow the optimal dose range and target populations as early as possible in clinical development could optimize exposure–response profiles and increase the implied power of the studies.^32^, ^33^, ^34^, ^35^ Likewise, using enriched populations, more likely to respond to treatment, can increase the implied effect size. However, this may come at the expense of generalizability to the intended target therapeutic population.
3. **Reducing variability**. Excessive variability in study population and execution of study procedures could decrease the power. Strict inclusion and exclusion criteria and precision in study execution both have the potential to increase the power of a study. Attention to the placebo effect and training to minimize its magnitude (e.g., by reducing expectation, nonspecific therapeutic effects, inflation of baseline values, and unblinding) will help reduce variability.^36^
4. **Use of repeated measures** has the potential to maximize power and increase the yield of available data and has been accepted by regulators as an alternative to the traditional Last Observation Carried Forward (LOCF) approach.^37^, ^38^, ^39^
5. **Bayesian statistical approaches** hold the promise of maximizing early‐phase clinical development by incorporating data from various nonclinical and clinical studies to reduce the uncertainties around study design and power calculation (e.g., effect size, dose ranges, study population).^17^, ^40^, ^41^, ^42^ The Bayesian approach, especially with sequential analysis with unlimited looks at the data with no penalty, could address some of the inherent uncertainties of early‐phase clinical development.
6. **Adaptive design** could enable early, seamless, and efficient selection of optimal doses (i.e., with the largest effect size), thus maximizing existing sample sizes for the study of the most effective doses. Adaptive design could also enable early termination of studies if convincing signals of efficacy or toxicity are identified early, thus mitigating some of the expenses of large sample size studies.^5^, ^16^, ^41^, ^43^, ^44^, ^45^
7. **Use of one‐tailed tests**. There have been calls to increase the power of clinical trials by including one‐tailed instead of two‐tailed significance levels in the analysis of the results. This, however, will allow testing of only the “side” of benefit while knowledge of the countereffects or harmful effects, also of public health and drug development relevance, will be missed (for example, if a drug for hypertension increased blood pressure instead of reducing it).
8. **Strategic approach**. Recent reports have introduced clinical development models and decision algorithms that could incorporate our conclusions to improve outcomes by adjusting the choice of error rates to the cost of the errors, and quantifying the corresponding successes and profits.^5^, ^46^, ^47^

A recent analysis by Lindborg *et al*. supports the general notion that higher‐powered early‐phase trials may increase treatment development productivity.^48^ Using different assumptions (e.g., a higher probability of success), methodology, and outcomes (e.g., using cost of development alone instead of including value of “false‐negatives”), they reach similar conclusions. They demonstrate that the values for alpha and beta that are optimal for treatment development productivity (alpha 0.15–0.35 and beta 0.05–0.15) differ from conventional values but resemble the results of our Scenario 4 (and in reversing the asymmetry of the Type‐I and Type‐II errors). However, while we demonstrate that increasing sample size at phase II is justified by the return on investment, Lindborg *et al*. suggest keeping the sample size the same as in traditional approaches. More detailed discussion of the similarities and differences between our analyses, and the combined implications to drug development strategies, is available in the **Supplemental Information (Additional Analyses**).

## Limitations and follow‐up analyses

Our analyses have several limitations and constraints. First, most of our assumptions (such as the cost of development, the determinants of developmental decisions, and details of sample size, effect size, and variability) depend on information that is often confidential, especially at the early stages of clinical development. Greater transparency among clinical development stakeholders is needed to enable informed research and consolidation of experience across sponsors. Second, there is a great deal of variability in developmental scenarios (e.g., use of one or two phase II studies) across therapeutic areas (e.g., in effect size and minimal meaningful clinical effect), types of treatment (e.g., small molecules vs. biologics), and costs of development. Our analyses and discussion therefore necessitated simplification of a complex developmental environment using assumptions that may not represent all existing scenarios. Third, our analyses and recommendations are limited to consideration of efficacy based on a simple hypothesis test. We acknowledge that equating “false‐positive” and “false‐negative” with alpha and 1‐power is an approximation, respectively, of their use in real practice, where a distribution of the effect size is normally used. We recognize that the real‐life relationship and interplay between the Type‐I and Type‐II errors can be complex and dependent on multiple factors, some possibly unknown at the time the studies were conducted, such as the probability of identifying poor disposition profile, signals of toxicity or intolerance, poor compliance, and dropouts. These could result in different “false‐positive” and “false‐negative” rates than predicted by the simple hypothesis test. Our calculations assume that a negative study (in terms of efficacy) always results in termination of a treatment from development. This may not always be the case, and even a weak statistical evidence of efficacy (e.g., *P* = 0.1) or evidence in a subgroup analysis may be sufficient to pursue development. The decision to eliminate a treatment from development is multipronged and depends not only on efficacy considerations but also on safety (including nonclinical toxicity and carcinogenicity data that may emerge during clinical development), pharmacokinetics, availability of resources, the profiles of other treatments in the pipeline, and strategic and competitive environment considerations. Our results should therefore be seen in the context of the role of efficacy as a determinant of treatment viability. Accordingly, we recommend that follow‐up analyses incorporate these aspects of developmental decision‐making and test our results across a range of therapeutic areas and stages of treatment development.

## SUMMARY

Early‐phase clinical studies are typically underpowered. In addition, an asymmetry between the two types of statistical errors means that “false‐negatives” are more likely to occur than “false‐positives,” are cumulative, and exit the development process upon discovery, preventing the verification of their “falseness” in higher‐powered follow‐up studies. The resulting “false‐negatives” mean loss and delay of effective treatments to patients and could be worth billions of dollars in untreated morbidity and mortality, and loss of commercial benefits to treatment developers. Our simulations provide information about the magnitude and correlates of the “false‐negatives” to support informed developmental decisions, and suggest that higher‐powered early‐phase studies are worth the investment. Our findings require replication, validation using a spectrum of therapeutic areas and developmental scenarios, and debate by the relevant treatment development stakeholders.

## Supporting information


**Supplemental Information**

**Supplemental Information**

**Supplemental Information**

**Supplemental Information**

**Supplemental Information**

**Supplemental Information**

**Supplemental Information. Box 1. Glossary of statistical Terms**

**Supplemental Information. Assumptions, Models, and Additional Analyses**
