# Supplementary figures and images for: The Burden of the “False‐Negatives” in Clinical Development: Analyses of Current and Alternative Scenarios and Corrective Measures
Source: Clin Transl Sci. 2017 Jul 4;10(6):470–9. doi: 10.1111/cts.12478 (PMC6402187; doi:10.1111/cts.12478)

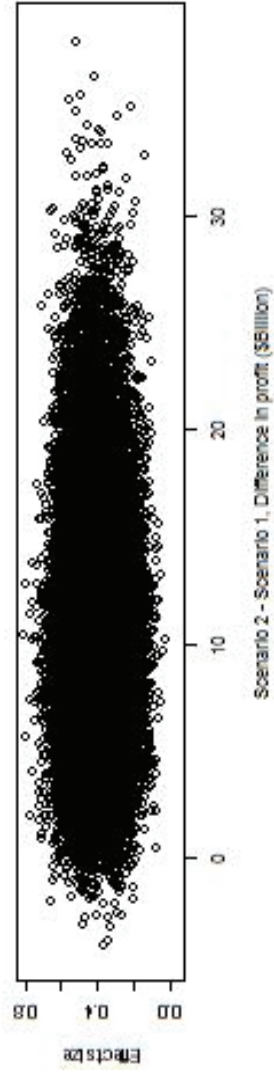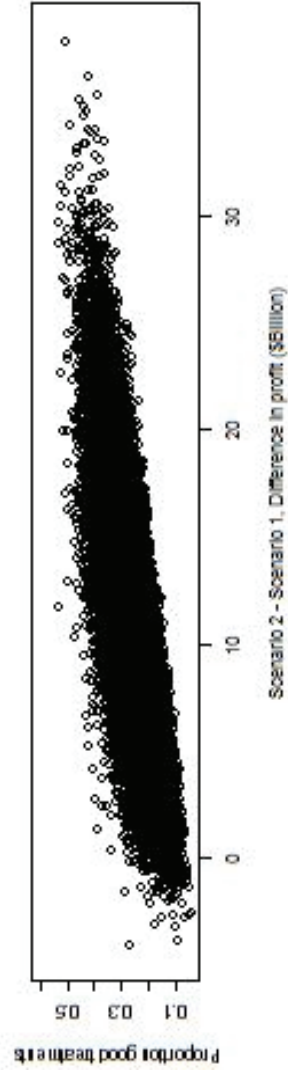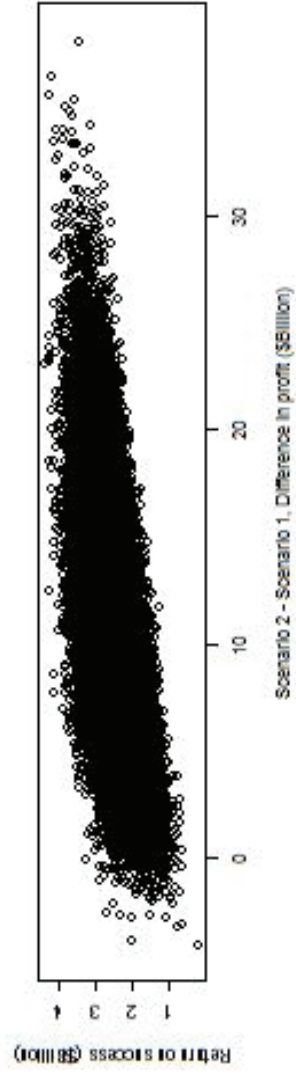

Supplement: Supplementary file 1 — Supplemental Information [file CTS-10-470-s001.pdf]

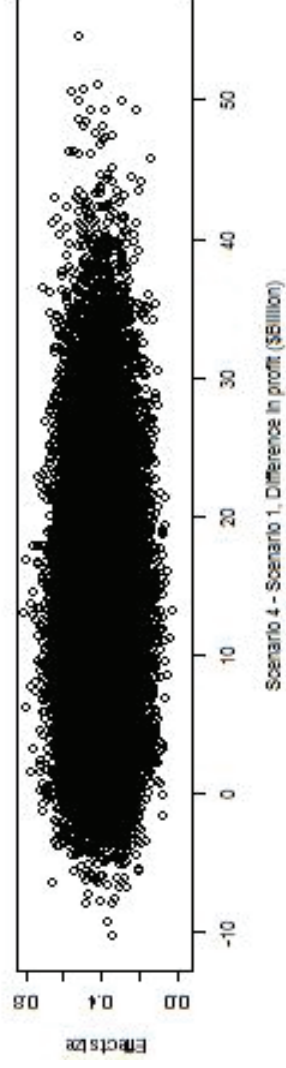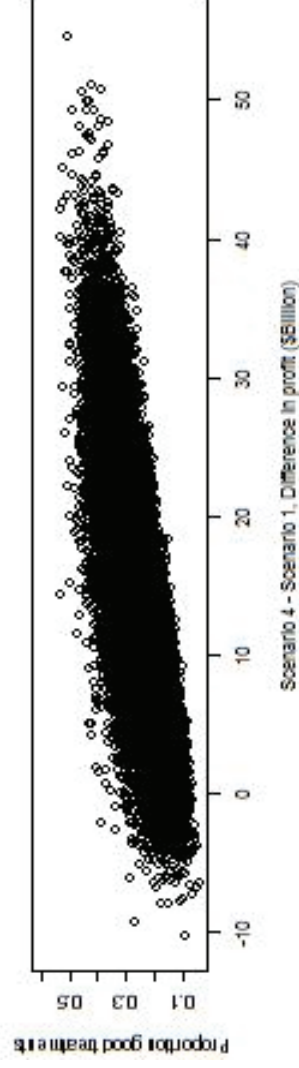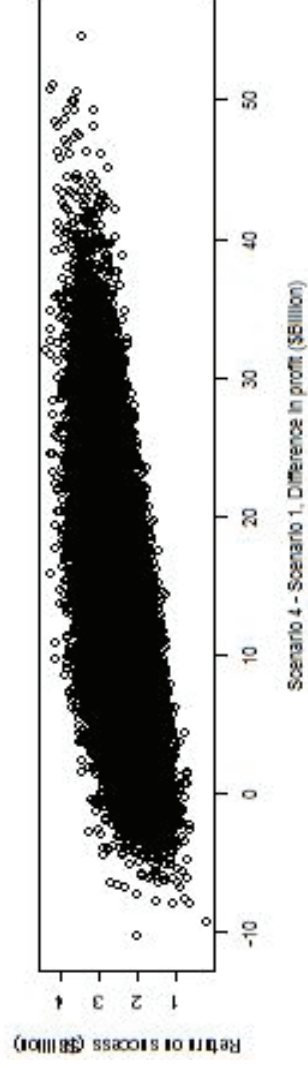

Supplement: Supplementary file 2 — Supplemental Information [file CTS-10-470-s002.pdf]
