# Supplementary material for: The Burden of the “False‐Negatives” in Clinical Development: Analyses of Current and Alternative Scenarios and Corrective Measures
Source: Clin Transl Sci. 2017 Jul 4;10(6):470–9. doi: 10.1111/cts.12478 (PMC6402187; doi:10.1111/cts.12478)
Supplement: Supplementary file 3 — Supplemental Information [file CTS-10-470-s003.docx]

| **Parameter** | **Distribution** | **Mean** | **Median** | **SD** | **95% CI** |
| --- | --- | --- | --- | --- | --- |
| **Effect size** | Normal | 0.4 | 0.4 | 0.1 | (0.2, 0.6) |
| **Good treatments** | Beta(10,30) | 0.25 | 0.25 | 0.067 | (0.13, 0.39) |
| **Scenario 1 Phase II Cost per patient** | Normal | 2.00E+05 | 2.00E+05 | 49922 | (1e+05, 3e+05) |
| **Return on success** | Normal | 2.50E+09 | 2.50E+09 | 5.00E+08 | (1.5e+09, 3.5e+09) |
