# Supplementary material for: The Burden of the “False‐Negatives” in Clinical Development: Analyses of Current and Alternative Scenarios and Corrective Measures
Source: Clin Transl Sci. 2017 Jul 4;10(6):470–9. doi: 10.1111/cts.12478 (PMC6402187; doi:10.1111/cts.12478)
Supplement: Supplementary file 4 — Supplemental Information [file CTS-10-470-s004.docx]

|  |  | **Mean** | **Median** | **SD** | **LL 95%** | **UL 95%** |
| --- | --- | --- | --- | --- | --- | --- |
| **Scenario 1** | **True Positives** | 10.13 | 9.95 | 2.74 | 5.29 | 15.94 |
|  | **False Negatives** | 14.88 | 14.62 | 4.02 | 7.78 | 23.41 |
|  | **False Positives** | 0.01 | 0.01 | 0.00 | 0.01 | 0.01 |
|  | **True Negatives** | 74.97 | 75.42 | 6.76 | 60.64 | 86.92 |
| **Scenario 2** | **True Positives** | 16.21 | 15.92 | 4.38 | 8.47 | 25.50 |
|  | **False Negatives** | 8.81 | 8.65 | 2.38 | 4.60 | 13.85 |
|  | **False Positives** | 0.01 | 0.01 | 0.00 | 0.01 | 0.01 |
|  | **True Negatives** | 74.97 | 75.42 | 6.76 | 60.64 | 86.92 |
| **Scenario 3** | **True Positives** | 10.13 | 9.95 | 2.74 | 5.29 | 15.94 |
|  | **False Negatives** | 14.88 | 14.62 | 4.02 | 7.78 | 23.41 |
|  | **False Positives** | 0.00 | 0.00 | 0.00 | 0.00 | 0.00 |
|  | **True Negatives** | 74.98 | 75.43 | 6.76 | 60.65 | 86.92 |
| **Scenario 4** | **True Positives** | 19.25 | 18.91 | 5.20 | 10.06 | 30.28 |
|  | **False Negatives** | 5.77 | 5.66 | 1.56 | 3.01 | 9.07 |
|  | **False Positives** | 0.04 | 0.04 | 0.00 | 0.03 | 0.04 |
|  | **True Negatives** | 74.95 | 75.39 | 6.75 | 60.62 | 86.88 |
