# Supplementary material for: The Burden of the “False‐Negatives” in Clinical Development: Analyses of Current and Alternative Scenarios and Corrective Measures
Source: Clin Transl Sci. 2017 Jul 4;10(6):470–9. doi: 10.1111/cts.12478 (PMC6402187; doi:10.1111/cts.12478)
Supplement: Supplementary file 5 — Supplemental Information [file CTS-10-470-s005.docx]

|  |  | **Mean** | **Median** | **SD** | **LL 95%** | **UL 95%** |
| --- | --- | --- | --- | --- | --- | --- |
| **Scenario 1** | **Costs** | 6.62 | 6.59 | 1.73 | 3.33 | 10.1 |
|  | **Return** | 25.3 | 24.4 | 8.6 | 11.2 | 44.5 |
|  | **Profit** | 18.7 | 17.9 | 8.39 | 4.84 | 37.4 |
| **Scenario 2** | **Costs** | 11.1 | 11.1 | 2.86 | 5.63 | 16.8 |
|  | **Return** | 40.5 | 39.1 | 13.8 | 17.8 | 71.2 |
|  | **Profit** | 29.4 | 28.0 | 13.5 | 6.92 | 59.6 |
| **Scenario 3** | **Costs** | 8.61 | 8.58 | 2.19 | 4.37 | 12.9 |
|  | **Return** | 25.3 | 24.4 | 8.60 | 11.1 | 44.5 |
|  | **Profit** | 16.7 | 15.9 | 8.55 | 2.51 | 35.7 |
| **Scenario 4** | **Costs** | 16.1 | 16.0 | 4.13 | 8.12 | 24.2 |
|  | **Return** | 48.2 | 46.5 | 16.3 | 21.2 | 84.7 |
|  | **Profit** | 32.1 | 30.6 | 16.1 | 5.39 | 68.1 |
