# Supplementary material for: The Burden of the “False‐Negatives” in Clinical Development: Analyses of Current and Alternative Scenarios and Corrective Measures
Source: Clin Transl Sci. 2017 Jul 4;10(6):470–9. doi: 10.1111/cts.12478 (PMC6402187; doi:10.1111/cts.12478)
Supplement: Supplementary file 6 — Supplemental Information [file CTS-10-470-s006.docx]

|  |  | **Mean** | **Median** | **SD** | **LL 95%** | **UL 95%** |
| --- | --- | --- | --- | --- | --- | --- |
| **Scenario 1** | **Costs** | 4.65 | 4.61 | 1.26 | 2.29 | 7.27 |
|  | **Return** | 25.22 | 24.32 | 8.56 | 11.21 | 44.72 |
|  | **Profit** | 20.57 | 19.60 | 8.27 | 6.98 | 39.40 |
| **Scenario 2** | **Costs** | 7.10 | 7.06 | 1.89 | 3.54 | 10.98 |
|  | **Return** | 40.34 | 38.89 | 13.70 | 17.92 | 71.53 |
|  | **Profit** | 33.24 | 31.67 | 13.32 | 11.35 | 63.54 |
| **Scenario 3** | **Costs** | 5.19 | 5.16 | 1.37 | 2.60 | 7.96 |
|  | **Return** | 25.20 | 24.29 | 8.56 | 11.19 | 44.69 |
|  | **Profit** | 20.01 | 19.06 | 8.34 | 6.28 | 38.90 |
| **Scenario 4** | **Costs** | 9.37 | 9.32 | 2.43 | 4.73 | 14.24 |
|  | **Return** | 47.97 | 46.26 | 16.27 | 21.35 | 85.01 |
|  | **Profit** | 38.60 | 36.75 | 15.95 | 12.40 | 74.85 |
