# Supplementary material for: The Burden of the “False‐Negatives” in Clinical Development: Analyses of Current and Alternative Scenarios and Corrective Measures
Source: Clin Transl Sci. 2017 Jul 4;10(6):470–9. doi: 10.1111/cts.12478 (PMC6402187; doi:10.1111/cts.12478)
Supplement: Supplementary file 7 — Supplemental Information. Box 1. Glossary of statistical Terms [file CTS-10-470-s007.docx]

**Box 1. Glossary of Statistical Terms**

***Statistical Significance.*** The probability of committing a Type I error (i.e., making a false positive decision). The level at which an event is statistically significant is known as the significance level. This is conventionally set to α = 0.05 (i.e., 5%). If the P-value of a test (the probability of obtaining a result at least as extreme) is lower than this threshold, the null hypothesis (e.g., that the treatment has no effect) is rejected in favor of the alternative hypothesis (e.g., that the treatment is efficacious).

***Statistical Power.*** The probability that a study will correctly reject the null hypothesis when the null hypothesis is false; that is, the probability of *not* committing a Type II error or making a false negative decision. The probability of committing a Type II error is referred to as the false negative rate (β), and power is equal to 1 – β. The power of a statistical test is also referred to as the sensitivity of the test.

***Type I Error / False Positive.*** A Type I error occurs when the null hypothesis is incorrectly rejected. In this case, a ‘bad’ treatment has been claimed efficacious, where in fact it has no beneficial effect.

***Type II Error / False Negative.*** A Type II error occurs when the null hypothesis is incorrectly *not* rejected (i.e., the null hypothesis is falsely accepted). In this case, an efficacious ‘good’ treatment has been falsely determined ineffective, often leading to termination of the development process. These are the ‘false negative’ treatments or ‘*missed opportunities*’.

***True Positives.*** Occurs when the null hypothesis is correctly rejected. In this case, a genuinely ‘good’ treatment is claimed to be efficacious. These ‘true positives’ are the ‘*successful treatments*’ of the development process.

***True Negatives.*** Occurs when the null hypothesis is correctly *not* rejected. In this case, a genuinely ‘bad’ treatment is *not* claimed to be efficacious. These are ineffective treatments that were appropriately eliminated.

***Effect Size.*** A standardized measure that quantifies the size of the difference between two groups or the strength of an association between two variables. As standardized measures, effect sizes allow estimates from different studies to be compared directly and also to be combined in meta-analyses.

***Phase 2 Studies.*** These are early-phase studies designed to test treatment efficacy and safety *in patients* following the safety and pharmacokinetic Phase 1 studies *in healthy volunteers*. Phase 2 studies are relatively small and short in duration, often testing around 100 – 300 patients for a few weeks or months. Treatments which are found to be efficacious and safe will continue to Phase 3.

***Phase 3 Studies.*** These larger, longer, confirmatory late-phase trials compare treatments found efficacious at Phase 2 with ‘standard-care’ treatments and/or placebo. Phase 3 studies are typically much larger (often by an order of magnitude) and longer than Phase 2 studies with sample sizes greater than 600 and often in the thousands.

***Last Observation Carried Forward (LOCF).*** A single imputation method for missing data in data sets with repeated measures. Each missing data point is replaced by the last observed value of that variable. This method is used to avoid loss of data where the analysis requires complete data. However, LOCF may introduce bias by overestimating precision (thus overestimating study power), and assigning intermediate efficacy to endpoints can lead to under-/over-estimates of efficacy if those who continue to improve / deteriorate, respectively, discontinue the study.

***Mixed-Methods Repeated Measures (MMRM).*** Obtaining repeated measures from individual patients (rather than just the last observation as in LOCF) reduces the variance and increases the amount of data about treatment effect, both of which increase the statistical power of the test for a given number of patients. Traditional repeated-measures designs assume sphericity (i.e., that all patients in the same group change in a constant way over repeated measures). Mixed-methods refer to statistical methods which allow for correlation of repeated measures within subjects *and* the comparison of subjects *between-groups* (e.g., treatment vs placebo), without assuming sphericity. That is, MMRM is more powerful as it can account for variations in follow-up times and missing data.

***Adaptive Designs.*** Trial design which uses pre-determined algorithm to modify study parameters (e.g., participant criteria, treatment dose, and sample size) based on intermediate analyses of accruing study data. Adaptive designs are used to increase study efficiency, speeding the identification of efficacious treatments, optimizing dosage, and identifying appropriate research participant groups.
