# Supplementary material for: The Burden of the “False‐Negatives” in Clinical Development: Analyses of Current and Alternative Scenarios and Corrective Measures
Source: Clin Transl Sci. 2017 Jul 4;10(6):470–9. doi: 10.1111/cts.12478 (PMC6402187; doi:10.1111/cts.12478)
Supplement: Supplementary file 8 — Supplemental Information. Assumptions, Models, and Additional Analyses [file CTS-10-470-s008.docx]

**Assumptions, Models, and Additional Analyses**

A probabilistic sensitivity analysis was conducted to test the robustness of our conclusions to variations in the input parameters. This analysis varied the effect size, the proportion of treatments that were actually effective, the costs per patient, and the return on success. The power and significance level, or alpha, of the scenarios was held fixed. Effect size was assumed to be normally distributed with mean 0.4 and standard deviation 0.1. As the proportion of treatments that were effective should be bounded between 0 and 1, it was assumed to follow a beta distribution, a common choice for modelling proportions. The parameters were selected as 10 and 30 to give a mean of 0.25 and 95% quantile of (0.13, 0.39), which were judged realistic by clinical opinion. The Scenario 1, or status quo, Phase-2 cost per patient was assumed to be normally distributed with mean $200,000 and standard deviation $50,000. All Phase-3 costs per patient were assumed to be 75% of the sampled Scenario 1 Phase-2 cost per patient as their larger sample sizes would benefit from economies of scale. The Scenario 2, 3 and 4 Phase-2 cost per patient were assumed to be 80% of the Scenario 1 Phase-2 costs as they also benefit from economies of scale, though to a lesser extent. The return on successfully approved treatment was assumed to be normally distributed with a mean $2.5 billion and standard deviation $0.5 billion. Full data and code, in the R statistical programming language version 3.1.2 (1), to implement this model and generate the results is provided below.

In addition, we conducted sensitivity analyses to explore greater effect sizes at Phase-2. We refer to these as ‘adjusted’ analyses (Table C.2). These were designed to account for the potential use of surrogate endpoints and/or enriched samples at Phase-2 trials that may result in greater effect size when compared with the clinically-relevant endpoints and/or non-enriched patient populations usually used at Phase-3. This of course is contingent on the validity of the surrogate endpoints and/or the enriched samples’ increased responsiveness to the therapeutic intervention, aspects that we did not factor into the analyses. In these ‘adjusted’ analyses, the sample size was first estimated using the same effect size as at Phase-3 (see the Discussion section). The effect at Phase-2 was then increased to achieve the same power as scenario 1 Phase-3 (90%) but with the smaller sample size that was initially estimated; a new Phase-2 sample size using the increased effect size was then calculated, which reduces the Phase-2 costs. As the significance level and power remain the same at phase-2, the number of successful treatments is the same. The impact of these adjustments is to reduce the costs at Phase-2 and therefore increase the final profit.

**The assumptions behind our analyses**

Our assumptions are derived from data available in the public domain and consistent with current staged-development scenarios (Scenario 1) such as the percentage of successful treatments entering Phase-2 (25%), Phase-2 power (50%), and percentages of treatments passing through Phase-2 and Phase-3 (16.3% and 61.9%, respectively) ([2](#_ENREF_2), [6-9](#_ENREF_6)). The key parameter of Phase-2 power of around 50% is supported also by the convention of using much smaller studies at Phase-2 than Phase-3, as acknowledged by the FDA: Phase-2 are “… conducted in a relatively small number of patients, usually involving no more than several hundred subjects”, while “Phase 3 studies usually include from several hundred to several thousand subjects” ([12](#_ENREF_12)).

To illustrate the impact of this sample size difference on study statistical power we use the following conservative example using similar numbers to those in our model. The sample size formula is ([13](#_ENREF_13)):

$$n= \frac{\left( z_{1-}+z_{1-} \right)^{2}\sigma^{2}}{\mu_{1}-\mu_{0}}$$

Where *n* is the sample size, *z* is the standardized normal distribution, is the Type-I error, is the Type-II error (*1-* is the power), *σ* is the standard deviation, and µ_1_-µ_0_ is the difference between the group means.

We assume, conservatively, a modest-sized Phase-3 study at (*n* = 672), and assume also that the study is adequately powered (at 90%) to detect the desired effect-size, with = 0.05, = 0.1 (i.e., power is 1 – 0.1 = 90%), µ_1_ = 1.9, µ_0_ = 1.7, *σ*  = 0.8. If the Phase-2 study is done with the conservatively large sample size of 246 the power is 50% assuming the other parameters (effect-size, variability, and Type-I error) remain the same between the phases. This is clearly under-powered with only 50% chance of detecting true effect. If the sample size of the Phase-2 study is 186 the power is only 40%.

Our assumptions are also consistent with the assumption that the prior probability of success (PPS) is higher in Phase-3 than in Phase-2 ([10](#_ENREF_10)) (25% PPS at Phase-2; 77% PPS at Phase-3 in Scenario 1; but even higher PPS at Phase-3 in Scenarios 2, 3, and 4). In other words, by the time a novel treatment enters Phase-3 there is a higher probability that it is an ‘effective’ treatment. This is also consistent with our finding that more, overall, is spent on Phase-2 programs than Phase-3 ($4,000 M rather than $2,646 M per 100 developmental programs, respectively), even though individual Phase-3 studies are more expensive than Phase-2 studies. This is explained by the larger number overall of Phase-2 studies due to large number of treatments failing at Phase-2, as has extensively been reported elsewhere ([2](#_ENREF_2), [7](#_ENREF_7), [14](#_ENREF_14)). We assumed same duration for Phase-2 and Phase-3 studies (2.5 years) even under the alternative scenarios with larger Phase-2 studies consistent with previous reports ([2](#_ENREF_2), [15](#_ENREF_15)). Developers have considerable flexibility and control over the duration of studies and can compensate for increased sample sizes by increasing the number of sites and/or speed of recruitment. The cost per participant ($200,000) is derived from the average cost of Phase-2 program ($40M) ([2](#_ENREF_2)) divided by the average number of subjects in Phase-2 studies (N = 200). In all cases, Scenario 4 (lenient alpha and higher power at Phase-2) performs the strongest, even when the cost per participant is doubled (Supplemental Information).

Under certain developmental scenarios it is plausible that regulators will consider a larger, higher-powered, Phase-2 study, as in our Scenarios 2 and 4, as one of the 2 confirmatory studies for market authorization. This is consistent with the 1997 Food and Drug Administration Modernization Act (FDAMA) that states “data from one adequate and well-controlled clinical investigation and confirmatory evidence… are sufficient to establish effectiveness”. This would obviously further increase the value of Scenarios 2 and 4 over the traditional Scenario 1 as it would spare the resources for an additional Phase-3 study. In such a case, our existing Scenarios 2 and 4 with their additional Phase-3 study should be considered conservative, and the conclusion, that these scenarios are more profitable than traditional scenario 1, robust to the assumption of eligibility for regulatory approval purposes. However, there are valid reasons not to consider larger Phase-2 studies as one of the 2 registration studies: duration of treatment (Phase-2 studies usually involve shorter duration of treatment than Phase-3), even higher power at Phase-3, optimal dosing and target population.

Exploration of correlation between simulated differences in profits under competing scenarios and sampled input parameters was also performed. It found clear correlation between difference in profits and both return on investment and proportion of good treatments and these maximized the superiority of Scenarios 2 and 4 over the status quo of Scenario 1 (Figures A and B). There appears to be little impact of sampled effect size on the differences in simulated profits.

**Differences and Similarities with the Lindborg et al. 2014 Publication**

A recent analysis by Lindborg et al. supports the general notion that higher-powered early-phase trials may increase treatment-development productivity ([16](#_ENREF_16)). Using different assumptions, methodology, and outcomes they demonstrate that the values for alpha and beta that are optimal for treatment-development productivity (alpha 0.15-0.35 and beta 0.05-0.15) differ from conventional values but resemble the results of our Scenario 4. That we reach similar results is a validation of both approaches in this complex scientific environment.

The strength of the Lindborg analyses is in the use of a comprehensive model (Paul et al. 2010) that spans the entire drug development process but they focus their analyses only on Phase-2. We used a more limited scope, focusing on the impact of power considerations, but included both Phase-2 and Phase-3, with more sensitivity analyses, greater detail in discussion, in a much larger and more comprehensive manuscript. Importantly, we used different methodology and assumptions as detailed below. Overall, we reach similar conclusions, but from rather different vantage points, and that convergence is important.

The Lindborg analyses do not consider the impact of the ‘false-negatives’ on loss of profit to developers, i.e., the topic of our manuscript – the burden of the ‘false-negatives’, but rather focuses on the cost per launch as the parameter reflecting productivity. In addition, the details of the productivity calculation/s that arrived at magnitude of reduction in cost per launch (6-7%), the key outcome of the analyses, are not provided. Our analyses, taking into account the value of ‘rescued’ ‘false-negatives’, suggest a much larger improvement in productivity (76.9% for Scenario 4). Lindborg et al. do acknowledge: “… more product launches would result, which would offset the Phase III spending”, however, no tangible analysis or quantification of the resulting benefit is provided. They also conclude “… intuitively it makes sense that the optimum ‘false-negative’ rate should be lower than the optimum FP rate, as FN mistakes are more costly”, however, again, no analysis is provided to support this intuition. Our analyses provide the quantitative results to support this intuitive conclusion.

Lindborg et al did not conduct sample size calculations to accompany the analyses of Type-II error, but conclude that the “improvement in productivity would not require more study patients…”. It appears that the only way proposed to increase the power is by increasing the Type-I error, effectively compensating reduction one error by increasing another. This does not increase the amount of data collected and the overall power of the study to detect meaningful differences between the null and alternative hypotheses. Our conclusion is that increase in sample size at Phase-2 is indeed worth the economic investment.

Other assumptions differ: We used a more conservative, lower probability, supported by a considerable body of literature of failure rates in the range of 30-40% for Phase-2 and 65-75% for Phase-3 for a combined average around 25% probability of efficacy at entry to Phase-2 while Lindborg et al. used 50%. This assumption is somewhat generous considering published success rates (DiMasi, Feldman et al. 2010, Paul, Mytelka et al. 2010, Scannell, Blanckley et al. 2012). We also provide results for 10% probability of efficacy. This core assumption had a considerable impact on the results.

Another difference is that Lindborg et al. assume alpha and beta values of 5% and 20% respectively, in both Phase-2 and phase-3 studies (Lindborg et al. 2014, Supplemental Information), whereas we assume that the current situation is far worse at Phase-2 - i.e., that power is at 50%. Our analyses use as a fundamental premise the ‘de facto’ under-power status of Phase-2 studies compared with the relatively adequate power of Phase-3. This is evidenced by the sample size differences, sometimes by an order or magnitude, between the two types of studies as indicated, for example, in FDA documents (FDA 2007). We have added a paragraph in the discussion section that illustrates the power differences simply as a function of sample size differences between Phase-2 and Phase-3. Other factors contributing to uncertainties at Phase-2 (e.g., regarding exposure-response correlations, and optimal target populations) further reduce the effective power of Phase-2 studies, so we think our choice of 50% power at Phase-2 is likely conservative.

Our manuscript focuses on sample size more closely, illustrating the effects of sub-optimal design in terms of cost based on numbers of participants. We provide sample size calculations for each phase in each scenario and the detailed economic productivity implications. The Lindborg analyses do not include discussion of sample size and only a rudimentary consideration of the economic implications of power scenarios. The productivity and sample size outcomes are presented only in figure format as shades of red and black, and dot size, respectively. Although multiple scenarios and their ranges are included in their analyses the conclusions provide only one rough estimate of the increase in productivity (6 - 10%). We believe we provide greater refinement and discrimination in our detailed quantitative results. We also suggest increased sample size as one of the options to increase power at Phase-2 while Lindborg et al recommend the sample size stays the same.

Lindborg et al. appear to not have run probabilistic sensitivity analyses (Monte Carlo component) so not have adequately represented the uncertainty in the assumptions underlying their model. Our Monte-Carlo analysis has allowed us to generate distributions and confidence intervals for effect size, proportion of ‘effective’ treatments at entry to clinical development, developmental costs, and expected returns. Lindborg only did simple deterministic analyses; this means they could only vary input parameters one at a time while all of ours varied simultaneously (which is important as the model function is nonlinear).

Finally, there are technical advantages to our manuscript. The Lindborg report is a short commentary (1500 words), with limited space to elaborate on methodology, results, and corrective measures. There is space for only 3 references to support the motivation, assumptions, and discussion. Even the Supplemental Information has limited information and multiple references to additional considerations that are not reported. Our supplemental Information provides the model code that could be used to apply, test, and improve our methodology. Lindborg et al. acknowledge the analyses are of a simple case but there is no further discussion of limitations. This important topic merits a more comprehensive discussion.

**Model Code**

The R code below implements the drug development model and Monte-Carlo analysis. The data and assumptions are included. It generates a file labelled “manuscript.results.matrix.csv” which is Table 2 in the main text. It also generates “parameter.summary.csv” which is Table A in this appendix, “clinical.results.matrix.csv” which is Table B, and “costs.results.matrix.csv” which is Table C.1 and Table C.2.

*# Monte Carlo analysis of drug development model*

*# Each scenario assumes 100 treatments go through Phase II/III trials*

*# Set the working directory of your choosing*

*baseline.directory<-"C:/XXX "*

*setwd(paste(baseline.directory,"/code",sep=""))*

*# Load package to perform sample size calculations*

*library(pwr)*

*# Number of samples*

*n.samples=10000*

*# Status quo (Phase II, Phase III parameter values)*

*# Alpha 0.05, 0.0025=(0.05*0.05)*

*# Beta 0.5, 0.19=1-(1-0.1)*(1-0.1)*

*scenario1<-matrix(c(0.05,0.5,0.05,0.1),ncol=2)*

*# Scenario 2, high power at Phase 2 (Phase II, Phase III parameter values)*

*# Alpha 0.05, 0.0025*

*# Beta 0.2, 0.19*

*scenario2<-matrix(c(0.05,0.2,0.05,0.1),ncol=2)*

*# Scenario 3, stringent alpha (Phase II, Phase III parameter values)*

*# Alpha 0.01, 0.0025*

*# Beta 0.5, 0.19*

*scenario3<-matrix(c(0.01,0.5,0.05,0.1),ncol=2)*

*# Scenario 4, lenient alpha*

*# Alpha 0.2, 0.25*

*# Beta 0.05, 0.19*

*scenario4<-matrix(c(0.2,0.05,0.05,0.1),ncol=2)*

*rownames(scenario1)<-rownames(scenario2)<-rownames(scenario3)<-rownames(scenario4)<-c("Alpha","Beta")*

*colnames(scenario1)<-colnames(scenario2)<-colnames(scenario3)<-rownames(scenario4)<-c("Phase II","Phase III")*

*# Cost of investigating n new drugs*

*# The sample size should be based on alpha, beta, effect.s*

*phase.cost<-function(scenario=1,adjust.phase2.effect=0,effect.s=0.4,cost.pp.1=100000,cost.pp.2=75000,success.return=(2500*10^6),good.t=0.25,n=100,alpha=NULL,beta=NULL)*

*{*

*# Base case assumes same effect size at phase II as at phase III*

*effect.s2<-effect.s*

*# Adjust phase II effect size based on scenario 1?*

*if(adjust.phase2.effect==1){*

*# Initial Sample size for Phase II*

*sample.size.adj<-tryCatch(200*pwr.t.test(d=effect.s,power=(1-scenario1[2,1]),sig.level=scenario1[1,1])$n/pwr.t.test(d=effect.s,power=0.5,sig.level=0.05)$n,error=function(e){10})*

*# Phase 2 effect size first assumed same as that at phase 3*

*effect.s2<-effect.s*

*# Use non-scaled sample size*

*sample.size.adj<-tryCatch(pwr.t.test(d=effect.s,power=(1-scenario1[2,1]),sig.level=scenario1[1,1])$n,error=function(e){10})*

*# Adjust Phase 2 effect size (keep sample size constant but increase power to that of Phase III (assumed 80%))*

*effect.s2<-pwr.t.test(n=sample.size.adj,power=0.8,sig.level=0.05)$d*

*}*

*# Extract scenario parameters and sample size*

*# Only set the alpha and beta if none were supplied to the function (is.null)*

*if(scenario==1)*

*{*

*if(is.null(alpha))alpha=scenario1[1,]*

*if(is.null(beta))beta=scenario1[2,]*

*}*

*if(scenario==2)*

*{*

*if(is.null(alpha))alpha=scenario2[1,]*

*if(is.null(beta))beta=scenario2[2,]*

*}*

*if(scenario==3)*

*{*

*if(is.null(alpha))alpha=scenario3[1,]*

*if(is.null(beta))beta=scenario3[2,]*

*}*

*if(scenario==4)*

*{*

*if(is.null(alpha))alpha=scenario4[1,]*

*if(is.null(beta))beta=scenario4[2,]*

*}*

*sample.size<-c(NA,NA)*

*# Keep alpha and beta (power) the same but different effect so sample size may be smaller*

*sample.size[1]<-tryCatch(200*pwr.t.test(d=effect.s2,power=(1-beta[1]),sig.level=alpha[1])$n/pwr.t.test(d=effect.s,power=0.5,sig.level=0.05)$n,error=function(e){10})*

*# Sample size for Phase III*

*sample.size[2]<-200*pwr.t.test(d=effect.s,power=(1-beta[2]),sig.level=alpha[2])$n/pwr.t.test(d=effect.s,power=0.5,sig.level=0.05)$n*

*# Two Phase III trials are conducted so double the sample size*

*sample.size[2]<-2*sample.size[2]*

*# Increase the alpha and beta appropriately*

*alpha[2]<-alpha[2]*alpha[2]*

*beta[2]<-1-(1-beta[2])*(1-beta[2])*

*# Matrix summarizing number of successes and failures at*

*# Phase II and Phase III*

*success.matrix<-matrix(NA,nrow=4,ncol=3)*

*rownames(success.matrix)<-c("Successful treatments","Missed opportunities","Approved placebos","Lucky escapes")*

*colnames(success.matrix)<-c("Phase II","Phase III","Total")*

*success.matrix[,1]<-c((1-beta[1])*good.t,beta[1]*good.t,alpha[1]*(1-good.t),(1-alpha[1])*(1-good.t))*n*

*# Good treatments at Phase III*

*success.matrix[1:2,2]<-c(1-beta[2],beta[2])*success.matrix[1,1]*

*# Bad treatments at Phase III*

*success.matrix[3:4,2]<-c(alpha[2],1-alpha[2])*success.matrix[3,1]*

*# Summary after both phases*

*# Successful treatments and approved placebos are results at Phase III*

*success.matrix[c(1,3),3]<-success.matrix[c(1,3),2]*

*# Missed opportunities and lucky escapes are sum of Phase II and III*

*success.matrix[c(2,4),3]<-success.matrix[c(2,4),1]+success.matrix[c(2,4),2]*

*# Work out the trial costs at Phase II and III*

*# Work out return from successful (approved) treatments and net profit*

*costs<-rep(NA,5)*

*names(costs)<-c("Phase II","Phase III","Total","Return","Profit")*

*costs[1]<-sample.size[1]*cost.pp.1*sum(success.matrix[,1])*

*costs[2]<-sample.size[2]*cost.pp.2*sum(success.matrix[,2])*

*# Per-patient Costs at Phase III are 0.75 those at Phase II*

*costs[2]<-0.75*costs[2]*

*# In scenarios 2 and 3, Phase II costs are 0.9 those in scenario 2 Phase II*

*if(scenario==2 | scenario==3 | scenario==4)costs[2]<-0.80*costs[2]*

*costs[3]<-sum(costs[1:2])*

*costs[4]<-sum(success.matrix[c(1,3),2]*success.return)*

*costs[5]<-costs[4]-costs[3]*

*return(list("sample.size"=sample.size,"success.matrix"=success.matrix,"costs"=costs,"effect.s2"=effect.s2))*

*}*

*phase.cost(scenario=2)#,alpha=c(0.05,0.003),beta=c(0.5,0.1))*

*phase.cost(scenario="x",alpha=c(0.5,0.05),beta=c(0.9,0.1))*

*# Function to summarise samples*

*summary.stats<-function(x,text.format=FALSE)*

*{*

*if(text.format)*

*{*

*return(paste(format(mean(x,na.rm=TRUE),digits=3)," (",format(quantile(x,probs=c(0.025),na.rm=TRUE),digits=3),", ",format(quantile(x,probs=c(0.975),na.rm=TRUE),digits=3),")",sep=""))*

*}else{*

*return(c(mean(x,na.rm=TRUE),median(x,na.rm=TRUE),sd(x,na.rm=TRUE),quantile(x,probs=c(0.025,0.975),na.rm=TRUE)))*

*}*

*}*

*# Sample some random effect sizes and returns on successful treatments*

*sample.parameters<-matrix(NA,ncol=5,nrow=n.samples)*

*colnames(sample.parameters)<-c("Effect size","Good treatments","Cost per patient II","Cost per patient III","Return on success")*

*sample.parameters[,"Effect size"]<-rnorm(n.samples,mean=0.4,sd=0.1)*

*# Beta distribution with mean 0.25 and (approximate) 95% range from 0.1 to 0.4*

*sample.parameters[,"Good treatments"]<-rbeta(n.samples,shape1=10,shape2=30)*

*sample.parameters[,"Cost per patient II"]<-rnorm(n.samples,mean=200000,sd=50000)*

*# Cost per patient at Phase III will be scaled by 0.75 or 0.9 depending on Phase II*

*sample.parameters[,"Cost per patient III"]<-sample.parameters[,"Cost per patient II"]*

*# Normal distribution for return on success with mean 2.5Billion and 99% interval from 1 to 4 billion*

*sample.parameters[,"Return on success"]<-rnorm(n.samples,mean=2.5*10^9,sd=0.5*10^9)*

*parameter.summary<-matrix(NA,nrow=5,ncol=4)*

*colnames(parameter.summary)<-c("Mean","Median","SD","95% CI")*

*rownames(parameter.summary)<-c("Effect size","Good treatments","Cost per patient II","Cost per patient III","Return on success")*

*for(i.parameter in 1:5)*

*{*

*parameter.summary[i.parameter,]<-summary.stats(sample.parameters[,i.parameter],text.format=TRUE)*

*}*

*write.csv(parameter.summary,file=paste(baseline.directory,"/results/parameter.summary.csv",sep=""))*

*# Need to record profits, costs, number of successful treatments, missed*

*# opportunities, approved placebos, lucky escapes*

*successful.treatments<-missed.opportunities<-approved.placebos<-lucky.escapes<-matrix(NA,nrow=n.samples,ncol=4)*

*scenario.returns<-scenario.costs<-scenario.profit<-array(NA,dim=c(2,n.samples,4))*

*phase2.costs<-phase3.costs<-array(NA,dim=c(2,n.samples,4))*

*for(i.sample in 1:n.samples)*

*{*

*for(i.scenario in 1:4){*

*# Loop over adjusted phase II effect size or not*

*for(i.adjust in c(2,1)) # End on unadjusted*

*{*

*i.phase.cost<-phase.cost(scenario=i.scenario,adjust.phase2.effect=(i.adjust-1),effect.s=sample.parameters[i.sample,"Effect size"],good.t=sample.parameters[i.sample,"Good treatments"],cost.pp.1=sample.parameters[i.sample,"Cost per patient II"],cost.pp.2=sample.parameters[i.sample,"Cost per patient III"],success.return=sample.parameters[i.sample,"Return on success"])*

*phase2.costs[i.adjust,i.sample,i.scenario]<-i.phase.cost$costs[1]*

*phase3.costs[i.adjust,i.sample,i.scenario]<-i.phase.cost$costs[2]*

*scenario.profit[i.adjust,i.sample,i.scenario]<-i.phase.cost$costs[5]*

*scenario.costs[i.adjust,i.sample,i.scenario]<-i.phase.cost$costs[3]*

*scenario.returns[i.adjust,i.sample,i.scenario]<-i.phase.cost$costs[4]*

*}*

*# Only the costs and returns depend on phase II effect size adjustment*

*successful.treatments[i.sample,i.scenario]<-i.phase.cost$success.matrix[1,3]*

*missed.opportunities[i.sample,i.scenario]<-i.phase.cost$success.matrix[2,3]*

*approved.placebos[i.sample,i.scenario]<-i.phase.cost$success.matrix[3,3]*

*lucky.escapes[i.sample,i.scenario]<-i.phase.cost$success.matrix[4,3]*

*}*

*}*

*# Report the means, medians, standard deviations, and 95% Credible intervals*

*results.matrix<-matrix(NA,nrow=4*4,ncol=5)*

*costs.results.matrix<-matrix(NA,nrow=2*4*3,ncol=5)*

*manuscript.results.matrix<-matrix(NA,nrow=4,ncol=5)*

*output.names<-c("Successful Treatments","Missed Opportunities","Approved Placebos","Lucky Escapes")*

*costs.output.names<-c("Costs","Return","Profit")*

*rnames<-costs.rnames<-c()*

*for(i.scenario in 1:4)*

*{*

*rnames<-c(rnames,paste("Scenario",i.scenario,output.names,sep=" "))*

*costs.rnames<-c(costs.rnames,paste("Scenario",i.scenario,costs.output.names,sep=" "))*

*}*

*costs.rnames<-c(costs.rnames,paste("Adjusted",costs.rnames))*

*rownames(results.matrix)<-rnames*

*rownames(costs.results.matrix)<-costs.rnames*

*colnames(results.matrix)<-colnames(costs.results.matrix)<-c("Mean","Median","SD","LL 95%","UL 95%")*

*rownames(manuscript.results.matrix)<-c("Scenario 1","Scenario 2","Scenario 3","Scenario 4")*

*colnames(manuscript.results.matrix)<-c("Phase II unadjusted","Phase II adjusted","Phase III","Profit unadjusted","Profit adjusted")*

*for(i.scenario in 1:4)*

*{*

*results.matrix[(i.scenario-1)*4+1,]<-summary.stats(successful.treatments[,i.scenario],text.format=FALSE)*

*results.matrix[(i.scenario-1)*4+2,]<-summary.stats(missed.opportunities[,i.scenario],text.format=FALSE)*

*results.matrix[(i.scenario-1)*4+3,]<-summary.stats(approved.placebos[,i.scenario],text.format=FALSE)*

*results.matrix[(i.scenario-1)*4+4,]<-summary.stats(lucky.escapes[,i.scenario],text.format=FALSE)*

*costs.results.matrix[(i.scenario-1)*3+1,]<-summary.stats(scenario.costs[1,,i.scenario],text.format=FALSE)*

*costs.results.matrix[(i.scenario-1)*3+2,]<-summary.stats(scenario.returns[1,,i.scenario],text.format=FALSE)*

*costs.results.matrix[(i.scenario-1)*3+3,]<-summary.stats(scenario.profit[1,,i.scenario],text.format=FALSE)*

*costs.results.matrix[4*3+(i.scenario-1)*3+1,]<-summary.stats(scenario.costs[2,,i.scenario],text.format=FALSE)*

*costs.results.matrix[4*3+(i.scenario-1)*3+2,]<-summary.stats(scenario.returns[2,,i.scenario],text.format=FALSE)*

*costs.results.matrix[4*3+(i.scenario-1)*3+3,]<-summary.stats(scenario.profit[2,,i.scenario],text.format=FALSE)*

*manuscript.results.matrix[i.scenario,1]<-summary.stats(phase2.costs[1,,i.scenario]/1000000,text.format=TRUE)*

*manuscript.results.matrix[i.scenario,2]<-summary.stats(phase2.costs[2,,i.scenario]/1000000,text.format=TRUE)*

*manuscript.results.matrix[i.scenario,3]<-summary.stats(phase3.costs[1,,i.scenario]/1000000,text.format=TRUE)*

*manuscript.results.matrix[i.scenario,4]<-summary.stats(scenario.profit[1,,i.scenario]/1000000,text.format=TRUE)*

*manuscript.results.matrix[i.scenario,5]<-summary.stats(scenario.profit[2,,i.scenario]/1000000,text.format=TRUE)*

*}*

*write.csv(results.matrix,file=paste(baseline.directory,"/results/clinical.results.matrix.csv",sep=""))*

*write.csv(costs.results.matrix,file=paste(baseline.directory,"/results/costs.results.matrix.csv",sep=""))*

*write.csv(manuscript.results.matrix,file=paste(baseline.directory,"/results/manuscript.results.matrix.csv",sep=""))*

**References**

(1) R Core Team. *R: A Language and Environment for Statistical Computing* (R Foundation for Statistical Computing: Vienna, Austria, 2015).

**Figure legends:**

**Figure A.** Correlation between Scenario 2 and Scenario 1. Input parameters: Effect size, proportion of good treatments, and return on success.

**Figure B.** Correlation between Scenario 4 and Scenario 1. Input parameters: Effect size, proportion of good treatments, and return on success.

**Table legends:**

**Table A. Simulation Assumptions.**

**Table B. Probabilistic simulation (Monte Carlo) analysis results.** Percent treatments in the ‘true positives’, ‘false negatives’, false positives’, and ‘true negatives’ categories for each of the scenarios.

**Table C.1. Economic Model. Costs, returns, and profits ($ Billions) in each of the 4 scenarios.**

**Table C.2. Economic Model. Costs, returns, and profits ($ Billions) in each of the 4 scenarios with adjusted effect size at Phase-2**
